# Metabolic maturation of differentiating cardiosphere-derived cells

**DOI:** 10.1016/j.scr.2021.102422

**Published:** 2021-07

**Authors:** Khadijeh Kathy Pakzad, Jun Jie Tan, Stephanie Anderson, Mary Board, Kieran Clarke, Carolyn A. Carr

**Affiliations:** a Department of Physiology, Anatomy & Genetics, University of Oxford, UK; b Advanced Medical and Dental Institute, Universiti Sains Malaysia, Penang, Malaysia

**Keywords:** Extracellular matrix, Glycolytic metabolism, Oxidative phosphorylation, Cardiac progenitor cells, Cardiomyogenic differentiation

## Abstract

Cardiosphere-derived cells (CDCs) can be expanded *in vitro* and induced to differentiate along the cardiac lineage. To recapitulate the phenotype of an adult cardiomyocyte, differentiating progenitors need to upregulate mitochondrial glucose and fatty acid oxidation. Here we cultured and differentiated CDCs using protocols aimed to maintain stemness or to promote differentiation, including triggering fatty acid oxidation using an agonist of peroxisome proliferator-activated receptor alpha (PPARα). Metabolic changes were characterised in undifferentiated CDCs and during differentiation towards a cardiac phenotype. CDCs from rat atria were expanded on fibronectin or collagen IV via cardiosphere formation. Differentiation was assessed using flow cytometry and qPCR and substrate metabolism was quantified using radiolabelled substrates. Collagen IV promoted proliferation of CDCs whereas fibronectin primed cells for differentiation towards a cardiac phenotype. In both populations, treatment with 5-Azacytidine induced a switch towards oxidative metabolism, as shown by changes in gene expression, decreased glycolytic flux and increased oxidation of glucose and palmitate. Addition of a PPARα agonist during differentiation increased both glucose and fatty acid oxidation and expression of cardiac genes. We conclude that oxidative metabolism and cell differentiation act in partnership with increases in one driving an increase in the other.

## Highlights

• Collagen IV promotes proliferation of cardiosphere-derived cells.
• Fibronectin supports differentiation of cardiosphere-derived cells.
• Oxidative metabolism increases as cardiac progenitors mature.
• Stimulating fatty acid oxidation promotes cardiac progenitor cell maturation.

## 1 Introduction

Cardiac mesenchymal cell therapy for myocardial infarction, such as that with cardiosphere-derived cells (CDCs), has yet to fulfil the hopes of the early protagonists, despite promising studies in animal models (Carr et al., 2011, Ellison et al., 2013, Noseda et al., 2015). In part, this is because expansion of progenitor cells from human tissue biopsies generates cells that may have been affected by disease and/or the insult to the heart and generation of sufficient numbers for therapy takes time, during which the damaged heart undergoes detrimental remodelling (Sutton and Sharpe, 2000). However, in most preclinical studies, cells are isolated and expanded from young healthy animals and administered at the time of surgical infarction. Understanding of the effect that expansion *in vitro* has on the cells can generate valuable information about the biology of the resident mesenchymal cell population and the conditions for optimum therapeutic culture.

Following isolation and expansion in high glucose media, cardiac progenitor cells are assumed to be glycolytic, yet the heart derives most of its energy needs from the oxidation of fatty acids (Taegtmeyer et al., 2005). Therefore, transition of non-contractile progenitor cells into beating cardiomyocytes requires transformation of the metabolic infrastructure, with mitochondrial network expansion and a switch from glycolysis to oxidative phosphorylation (Malandraki-Miller et al., 2018). Such a switch occurs in differentiating pluripotent stem cells (Chung et al., 2010, Lopez et al., 2021) and in isolated cardiac mesenchymal cells from the mouse heart (Malandraki-Miller et al., 2019).

In the developing heart, the extracellular matrix (ECM) provides cues to guide cell proliferation, migration and differentiation, with changes in ECM composition affecting tissue development and maintenance (Hanson et al., 2013). Among the most prevalent, functionally relevant, ECM proteins in the developing heart, collagen IV (ColIV) is found in atrial stem cell niches, whereas collagen I, which provides structural support, and fibronectin (FN), which mediates changes in the cellular phenotype, are found outside the niche in the myocardium (Schenke-Layland et al., 2011). ColIV induces differentiating embryonic stem cells (ESCs) to form early-stage cardiac progenitors and enhances their expansion whereas FN promotes the differentiation of ESC-derived progenitors into cardiomyocytes (Schenke-Layland et al., 2011). We postulated that expansion of cardiosphere-derived cells (CDCs) on ColIV would generate sufficient cardiac progenitors for therapy more quickly than expansion on FN. Furthermore, since cardiospheres form more rapidly in hanging drops, as the cells aggregate under gravity, we inferred this would maintain stemness as the cells would spend longer in the hypoxic core of the sphere. In parallel, we hypothesised that differentiation of cells on fibronectin might be more efficient than that on collagen IV and that by selection of culture conditions we could generate CDCs at different stages of differentiation to the cardiac phenotype. We compared the metabolic characteristics of CDCs cultured via the two protocols to determine whether oxidative metabolism is activated in progenitor cells during differentiation and whether that is a gradual process or induced only as differentiated progenitors mature. Finally we aimed to further upregulate fatty acid oxidation and mature the metabolic phenotype of the cells by stimulating the peroxisome proliferator-activated receptor alpha (PPAR α) pathway using the agonist WY-14643 (Gentillon et al., 2019).

## 2 Materials and methods

### 2.1 Animals

Male Sprague Dawley (SD) male rats were obtained from a commercial breeder (Harlan, Oxon, UK). Animals were kept under controlled conditions for temperature, humidity and light, with water and rat chow available ad libitum. Rats were anaesthetised with sodium pentobarbital (270 mg/kg body weight, IP; Euthatal, Merial, UK) to allow tissue removal. All procedures were approved by the University of Oxford Ethical Review Committee in accordance with Home Office (UK) guidelines under The Animals (Scientific Procedures) Act, 1986 and with University of Oxford, UK institutional guidelines.

### 2.2 Isolation, and expansion of cardiosphere-derived cells

Rat CDCs were cultured as previously described, with culture on fibronectin-coated plates and cardiosphere formation in non-adherent poly-d-lysine coated plates (Tan et al., 2011) or with culture on collagen IV coated plates and cardiosphere formation in hanging drops. Briefly, SD rat hearts (5 weeks old) were excised and hearts weighed (n = 6). Atrial and apex tissues were minced into 1 mm^3^ explant fragments in 0.05% trypsin-EDTA (Invitrogen). Explants were plated in petri dishes precoated with either 4 µg/ml fibronectin from bovine plasma (Sigma) (Fig. S1a) or 10 µg/ml collagen IV from Engelbreth-Holm-Swarm murine sarcoma (Sigma) (Fig. S1f). Complete explant medium (CEM; Table S1) was added and cells were incubated at 37 °C in 5% CO_2_. Supporting cells and phase bright cells (collectively known as explant-derived cells, EDCs), which had grown out from the explants, were harvested and resuspended in cardiosphere growth medium [CGM, Table S1] at a density of 3 × 10^4^ cells per well of 24 well plates precoated with poly-d-lysine (Fig. S1b) or 1000 cells per 25 µl drop in the hanging drop technique (Fig. S1g) and cultured for 4 days (Figs. S1b-c and S1g-h). Cardiospheres were subsequently expanded in CEM on FN or ColIV-coated tissue culture flasks (Figs. S1d-e or S1i-j, respectively) to generate cardiosphere-derived cells (CDCs), which were maintained in culture with CEM changed every 3 days and passaged every 7 days until passage 2 (P2). All experiments in this study used P2-CDCs at 70 to 80% confluency, unless otherwise stated. At the end of each stage (EDCs, P1-CDCs, and P2-CDCs), confluent cells were enzymatically digested, and counted using a haemocytometer under an inverted light microscope (Nikon, UK). The cell number was calculated per milligram tissue explanted.

### 2.3 Cell proliferation assay

P2-CDCs (250–20,000 cells per well) were seeded in 96-well plates pre-coated with either FN or ColIV. On days 1, 2, 3, 5, and 7, culture media was aspirated and cells were washed with PBS and kept at 80 °C. Proliferation was assessed using CyQUANT® Cell Proliferation Assay Kit (Molecular Probes, Invitrogen) plus RNase treatment, according to the manufacturer’s instructions. In brief, frozen cells were thawed at room temperature, treated with RNase, and lysed by addition of a buffer containing the CyQUANT GR dye. Fluorescence was then measured directly using a microplate reader (FluoStar, BMG) with excitation at 485 ± 10 nm and emission detection at 530 ± 12.5 nm. The assay was calibrated using a standard curve of bacteriophage λ DNA standard according to the manufacturer’s instruction.

### 2.4 Cardiomyogenic differentiation

P2-CDCs (10,000/cm^2^) were seeded on either FN or ColIV coated plates containing cardiomyogenic differentiation medium-1 (CDM-1; Table S1) and were treated with 5-Azacytidine (5-Aza) for 3 days, followed by ascorbic acid (AA; Sigma, UK) from day 6–14 (Fig. 2A). For later experiments, P2-CDCs (20,000/cm^2^) created through CoIV/HD culture, were seeded in CEM on plates coated with 1 mg/ml FN in 0.01% gelatin. Once cells reached confluency, cardiomyogenic differentiation medium-2 (CDM-2 Table S1) containing 5-Aza (5 µM) was added for 3 days. On day 4, the medium was changed for CDM-2 only without 5-Aza and refreshed every 2–3 days until day 7. On day 7 medium was replaced with one of the following media: TGFβ: CDM-2 containing 1 ng/ml TGF-β (Peprotech, UK) and 0.1 mM AA; or WY-14643: CDM-2 containing 1 ng/ml TGF-β and 0.1 mM AA plus 100 µM WY-14643 (PPARα agonist, Sigma, UK), each alternating with CDM-2 containing 0.1 mM AA until day 30 as shown in Fig. 5A. Cytotoxicity testing for 5-Aza and WY-14643 is shown in Fig. S3.

### 2.5 Flow cytometry

Control and differentiated CDCs were digested to a single cell suspension using 0.05% trypsin (5 min at 37 °C), washed with PBS, and fixed with 4% paraformaldehyde (Sigma) for 10 min. For intracellular markers, the cells were permeabilised with 0.2% Triton X-100 in PBS for 10 min. The samples were incubated with blocking solution (2% bovine serum albumin, BSA) + 2% fetal bovine serum (FBS) in PBS for 30 min and then treated with primary antibodies (Table S2) for 1 h. After rinsing with PBS, cells were treated with the secondary antibodies (Table S2) for 30 min, then washed and resuspended with fluorescence-activated cell sorting (FACS) buffer (DPBS with 0.5% BSA). and analysed using a FACS Calibur Flow Cytometer (BD Biosciences, San Jose, CA). The data were analysed using FlowJo version 10.7 (Fig. S2).

### 2.6 Confocal microscopy

CDCs grown on cover slips were fixed with PFA, immuno-labelled with primary then secondary antibodies for stem cell markers (c-Kit, CD90) or cardiac specific markers (cTnnT2, MHC, MLC, CXC43, and Titin) and scanned using an Inverted Olympus FV1000 Confocal system. Rat neonatal cells were stained for cardiac proteins as a positive control (Fig. S4) and differentiated CDCs were stained with secondary antibodies only as a negative control (Fig. S5). Organoids were imaged as a Z-stack and optical sections merged to give a 3D reconstruction of the full length of the sample.

### 2.7 RNA extraction and Real-Time quantitative reverse transcriptase PCR

Total RNA was extracted and purified from control or differentiated CDCs using a QIAGEN RNeasy Mini Kit (QIAGEN, United Kingdom). RNA concentration and purity were determined by measuring the absorbance at 230, 260, and 280 nm, using a NanoDrop spectrophotometer (NanoDrop technologies, Wilmington, USA). Complementary DNA (cDNA) was synthesized from RNA template using the AB high-capacity reverse transcriptase kit (Applied Biosystems, Paisley, UK) according to the manufacturer’s instructions. Primer pairs were designed based on interpretation of GeneBank or Ensemble Genome Browser and Integrated DNA Technology (IDT) (Table S3). Primer specificity was enhanced by designing a primer pair that flanked the exon-exon border of the gene of interest. Primer specificity was confirmed by BLASTing the primer sequence against genomic databases available at NCBI and primer amplification efficiency tests. Real-time PCR amplification was performed using the Applied Biosystem StepOnePlus Real-time PCR system (AB 44 International, Canada) with postamplification melting curves acquired to verify the specificity of PCR products. Relative quantification of target gene expression, normalized to the geometric mean of the housekeeping genes β-Actin (Actb) and β-2 microglobulin (β2M) and one calibrator (the control sample), was performed using comparative Ct method (the 2-ΔΔCt method).

### 2.8 Determination of glycolytic flux

Glycolysis was measured as the production of ^3^H_2_O from 5-^3^H-glucose. P2-CDCs (20,000/cm^2^) were seeded on pre-coated 6-well plates coated with either FN or ColIV and containing CEM. After overnight incubation to allow the cells to attach or at the end of the differentiation procedure, cells were given 10 ml of control or cardiomyogenic differentiation medium spiked with a trace of D[5-^3^H]-glucose (1.48 MBq/mmol; Amersham, Bucks, UK). The glycolytic rates were determined by collecting 0.6 ml of aliquots of culture medium after 8 h. ^3^H_2_O was separated from ^3^H-glucose using a Dowex anion exchange column (Sigma, UK) and read using a Tri-Carb 2800TR Liquid Scintillation Analyser 28.

### 2.9 Determination of substrate oxidation rates

Based on the method of Collins et al (Collins et al., 1998), with some modification, control or differentiated CDCs, in a 6-well plate, were incubated for 4 h in the presence of a single substrate spiked with radiolabelled tracer in DMEM (containing no pyruvate, glucose or glutamine) in a total volume of 1 ml/well (Board et al., 2017). Substrates tested were: 10 mM glucose containing 21 MBq/mmol D-U-^14^C-glucose; 5 mM pyruvate containing 0.35 MBq/mmol 1-^14^C-pyruvate; 10 mM acetoacetate containing 0.185 MBq/mmol 3-^14^C-acetoacetate; 2 mM glutamine containing 1.2 MBq/mmol U-^14^C-glutamine and 2 mM palmitate containing 10.27 Mβq/mmol 1-^14^C-palmitate. In addition, wells without cells containing DMEM with ^14^C-substrate were used as a background radioactivity control. The ^14^CO_2_ produced was trapped on KOH-soaked filter papers covering the inside of the 24-well plate lid of the apparatus (Fig. S6). A perforated rubber gasket, with holes corresponding to each well of the 24-well plate, separated the two plates. Perchloric acid was added to the well, at each desired time-point, to kill the cells and the plates were gently agitated for 60 min to release dissolved ^14^CO_2_. Filter papers containing trapped ^14^CO_2_ were counted in Ecoscint (National Diagnostics) using a Tri-Carb 2800TR Liquid Scintillation Analyser.

### 2.10 Calculation of ATP production

ATP yields were calculated assuming full oxidation of each substrate: glycolysis, 2 mol ATP/mol glucose; glucose oxidation, 31 mol ATP/mol glucose; acetoacetate oxidation, 20 mol ATP/mol acetoacetate; glutamine oxidation, 20 mol ATP/mol glutamine; palmitate oxidation, 106 mol ATP/mol palmitate.

### 2.11 Statistical analysis

Data are presented as mean ± SEM. Data were analysed using an unpaired TTest or ANOVA (GraphPad Prism v8.0.1) with a TUKEY multiple comparison as appropriate.

## 3 Results

### 3.1 The effect of extracellular matrix on isolation and expansion of CDCs

**Fig. 1:**
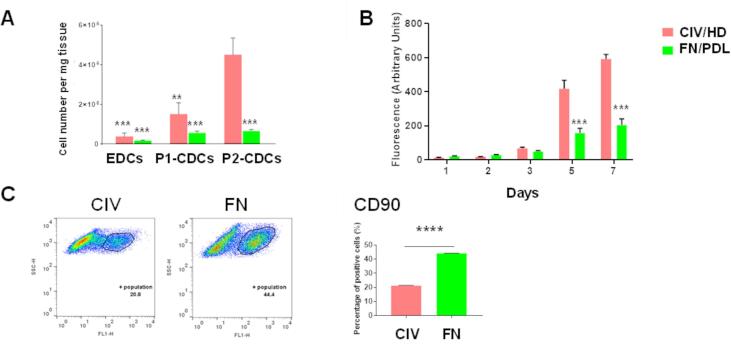
**Characterization of cardiosphere-derived cells. (**A) Yield of explant-derived cells and CDCs at P1 and P2 for cells cultured on FN/PDL or ColIV/HD (n = 6). (B) Proliferation of control P2-CDCs on FN or ColIV (n = 3). Data are presented as mean ± SEM and assessed using a two-way ANOVA with Tukey post hoc test ** p < 0.01, *** p < 0.001 compared with P2-CDCs on ColIV for (A) and with P2-CDCs on ColIV at the same time point for (B). (C) Representative flow cytometry dot plots and quantification of flow cytometry analysis of P2-CDCs for CD90 Data are presented as mean ± SEM (n = 3) and assessed using an unpaired *t*-test. **** p < 0.0001.

Adult cardiac stem/progenitor cells were isolated and expanded using the traditional method on fibronectin (FN) with cardiosphere formation on poly-D-lysine (PDL) (Fig. S1a-e) or using a modified technique of expansion on collagen IV (ColIV) with cardiosphere formation in hanging drops (HDs) (Fig. S1f-j). Explant-derived cells formed cardiospheres more quickly in hanging drops than on PDL (Fig. S1b, S1g) and formed larger spheres by day 4 (Fig. S1c, S1h). Culture on ColIV/HD resulted in significantly more cells at each stage of expansion and in a 10-fold increase in the number of CDCs at passage 2 (P2-CDCs, p = 0.0004 vs cells expanded on FN) after 46 ± 5 days (Fig. 1A). In addition, P2-CDCs created through ColIV/HD culture had significantly higher proliferation rates in comparison to those on FN/PDL (p < 0.0001; Fig. 1B), indicative of a more stem-cell like phenotype. Flow cytometry revealed that P2-CDCs created through both techniques expressed CD90 (Fig. 1C) with few cells expressing c-Kit, Oct4 or Nanog (data not shown) However, immunocytochemistry for c-kit on CDCs expanded on FN (Fig. S7) showed that the protein was expressed but had been internalized and therefore the cells would not have shown positive with flow. Both populations contained <5% of cells expressing the cardiac transcription factor, Nkx-2.5, the cardiac specific proteins, cardiac troponin T (cTnnT2), Titin and cardiac myosin heavy chain (cMHC), and the endothelial cell marker, von Willebrand factor (vWf) (data not shown). CDCs cultured on ColIV had significantly lower expression of CD90 than those cultured on FN..

### 3.2 The effect of extracellular matrix on differentiation of CDCs

**Fig. 2:**
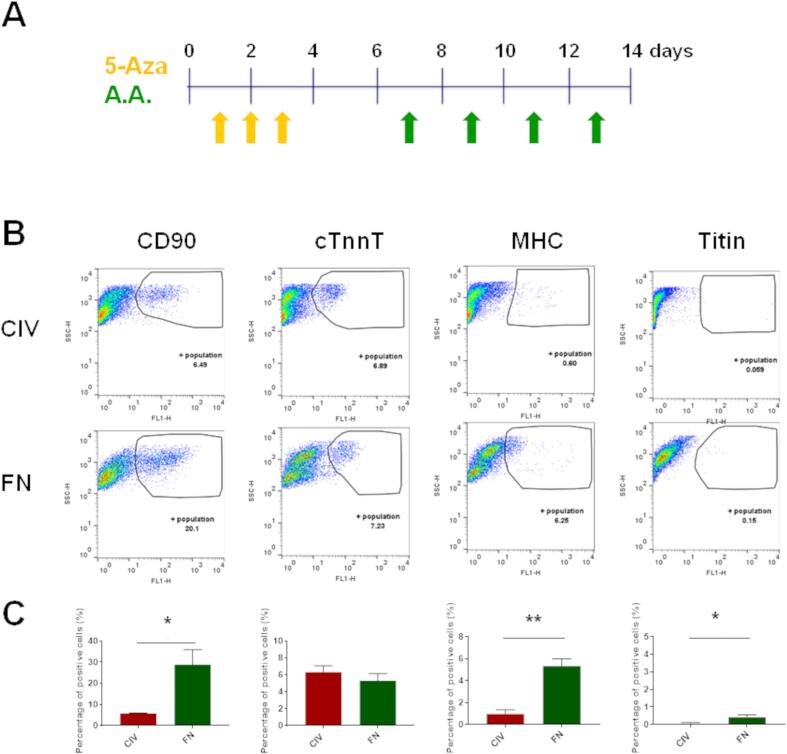
**Cardiomyogenic differentiation of passage-2 cardiosphere-derived cells.** (A) Timeline of the differentiation procedure showing days of addition of 5 µM 5-Aza (yellow arrows) or 0.1 µM ascorbic acid (green arrows) to the differentiation medium, which was refreshed every 2–3 days. (B) Representative flow cytometry dot plots and (C) quantification of flow cytometry analysis of cells expressing CD90, cTnnT2, cMHC and titin in P2-CDCs differentiated on CollIV or FN. Data are presented as mean ± SEM (n = 3) and assessed using an unpaired *t*-test. * p < 0.05; ** p < 0.01. (For interpretation of the references to colour in this figure legend, the reader is referred to the web version of this article.)

**Fig. 3:**
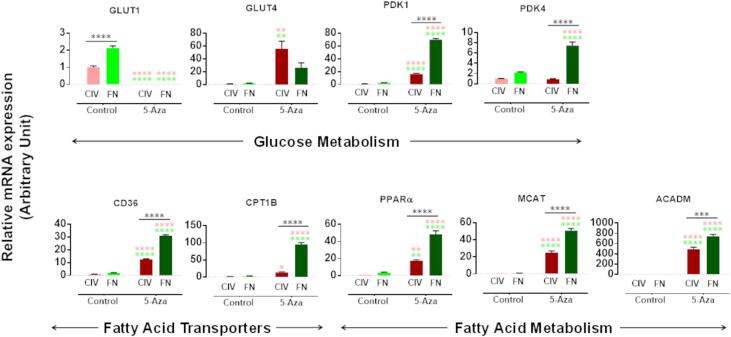
**Expression of metabolic-related genes in control and 5-Aza treated CDCs.** The relative mRNA expression of genes involved in glucose (GLUT1, GLUT4, PDK1 and PDK4) and fatty acid (CD36, CPT1B, PPARα, MCAT and ACADM) metabolism was assessed using qPCR in control and differentiated P2-CDCs created through FN/PDL or ColIV/HD. The mRNA expression was normalized to the geometric mean of Actb and β2M as reference genes and ColIV control as calibrator. Data are presented as mean ± SEM (n = 3), assessed using an ANOVA with Tukey post hoc test. Multicolour stars indicate multiple comparisons between groups and FN and ColIV control, black stars indicate comparison between control and differentiated cells. *p < 0.05; **p < 0.01; ***p < 0.001; **** p < 0.0001.

Induction of cardiomyogenic differentiation using 5-azacytidine (5 Aza) and ascorbic acid (AA) (Fig. 2A) resulted in a small number of cells expressing the cardiac specific proteins, cTnnT2, cMHC and Titin. Flow cytometry suggested that the larger cells in the population were those that began to express cardiac proteins, with significantly greater expression of MHC and titin in cells cultured on FN (Fig. 2B). Representative images of 5-Aza-treated cells grown on FN are shown in Fig. S8. Although some treated CDCs expressed cTnnT, αSA, cMyHC and cTnnI, they did not form beating cardiomyocytes and did not show the structural features such as sarcomeres seen in neonatal and adult cardiomyocytes (see eg Fig. S4). Treatment with 5-Aza induced a decrease in mRNA expression of glucose transporter 1 (GLUT1) to below the level of detection in the whole cell population (Fig. 3) and increased expression of pyruvate dehydrogenase kinase isozyme 1 and 4 (PDK1 and PDK4), which control the flux through pyruvate dehydrogenase to mitochondrial oxidation. In addition, there was an increase in mRNA expression of the fatty acid transporters, fatty acid translocase (CD36) and carnitine palmitoyltransferase IB (CPT1B), and of peroxisome proliferator-activated receptor α (PPARα), malonyl CoA-acyl carrier protein transacylase (MCAT) and acyl-coenzyme A dehydrogenase (ACADM) (Fig. 3). GLUT4 expression level was very low in untreated cells, but expression increased 25-fold after 5-Aza treatment on ColIV. For all other genes tested, expression was significantly greater in cells grown on FN than in those on ColIV (Fig. 3). Western blot analysis of levels of glucose transporters and mitochondrial proteins was less conclusive with few significant differences (Fig. S9).

### 3.3 Substrate metabolism and ATP production in control and differentiated CDCs

**Fig. 4:**
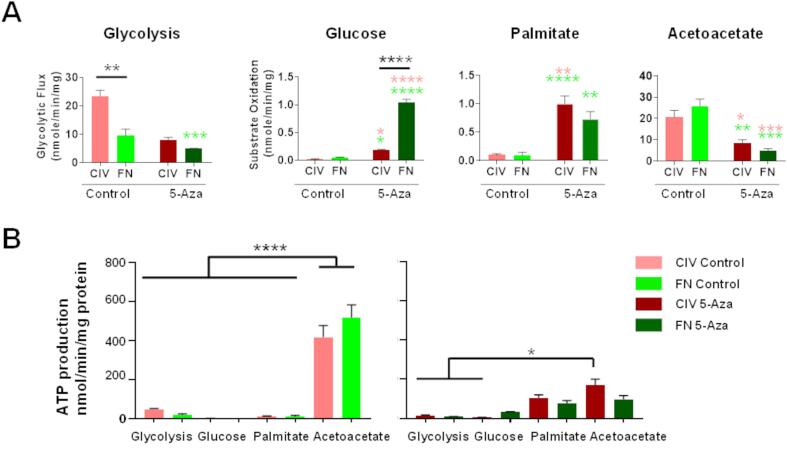
**Substrate utilisation by control and 5-Aza treated CDCs**. (A) The rate of glycolysis and of oxidation of glucose, palmitate and acetoacetate measured in control and differentiated P2-CDCs created through FN/PDL or ColIV/HD. (B) Calculated rates of adenosine triphosphate (ATP) production in control and differentiated P2-CDCs. Data are presented as mean ± SEM (n = 3–5), assessed using an ANOVA with Tukey post hoc test. Multicolour stars indicate multiple comparisons between groups and FN and ColIV control, black stars indicate comparison between control and differentiated cells for A and as indicated for B; * p < 0.05; ** p < 0.01; *** p < 0.001; **** p < 0.0001.

Substrate metabolism was assessed in cells cultured in 10 mM glucose, 10 mM acetoacetate, 2 mM glutamine or 2 mM palmitate. Glycolytic flux was considerably higher in P2-CDCs cultured on ColIV than in those cultured on FN (ColIV 23.4 ± 2.0, FN 9.6 ± 2.2 nmol/min/mg), but decreased after 5-Aza treatment (ColIV 8.0 ± 0.8, FN 5.0 ± 0.1 nmol/min/mg; Fig. 4A). Interestingly untreated cells oxidised acetoacetate at a significantly higher rate than glucose or palmitate (ColIV 20.8 ± 3.0, FN 25.8 ± 3.2, nmol/min/mg for Acetoacetate; ColIV 0.02 ± 0.015, FN 0.05 ± 0.01 nmol/min/mg for glucose; ColIV 0.1 ± 0.02, FN 0.09 ± 0.05 nmol/min/mg for palmitate; Fig. 4A). Oxidation of acetoacetate decreased after 5-Aza treatment whilst that of glucose and palmitate increased (ColIV 8.5 ± 1.5, FN 5.0 ± 0.1, nmol/min/mg for acetoacetate; ColIV 0.2 ± 0.02, FN 1.0 ± 0.06 nmol/min/mg for glucose; ColIV 1.0 ± 0.1, FN 0.7 ± 0.1 nmol/min/mg for palmitate; Fig. 4A).

Glucose oxidation after 5-Aza treatment was significantly higher in cells grown on FN than in those on ColIV (p < 0.0001; Fig. 4A). Glutamine oxidation was <0.2 nmol/min/mg in control cells from both conditions and did not change after 5-Aza treatment (data not shown). Palmitate oxidation increased significantly after treatment but occurred at comparable rates in cells cultured on ColIV and FN. The increased mitochondrial respiration in differentiated cells was supported by staining for the mitochondrial membrane potential using Mitotracker Red CMXRos (Fig. S10). The rate of ATP production was calculated based on the glycolytic flux and oxidation rates. P2-CDCs generated more ATP by oxidation of acetoacetate than from other substrates (p < 0.0001; Fig. 4B). Acetoacetate oxidation decreased after 5-Aza treatment such that ATP production in cells cultured on FN by oxidation of acetoacetate was comparable to that from other substrates whereas that from cells cultured on ColIV remained significantly higher than from glycolysis or glucose oxidation in those cells.

### 3.4 Stimulating fatty acid oxidation by pharmacological upregulation of the PPARα pathway

**Fig. 5:**
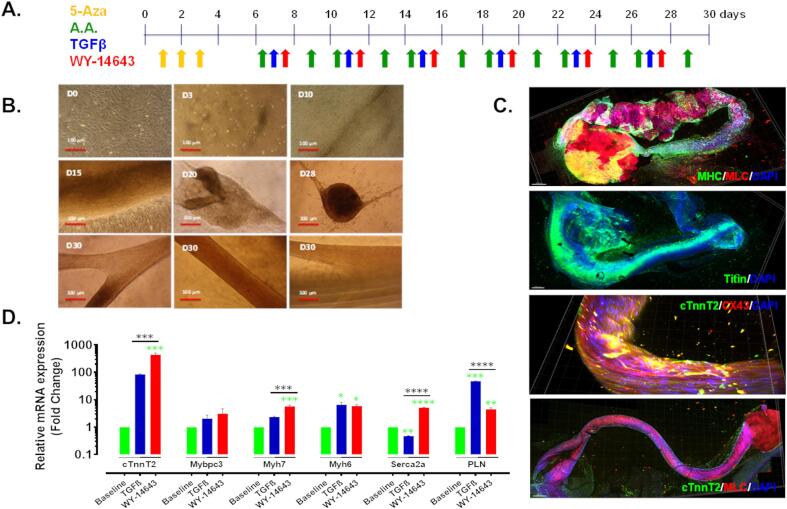
**Differentiated and WY-14643 treated cells form organoids.** (A) CDCs, expanded on ColIV were plated on FN and differentiated by treatment with 5-Azacytidine (5-Aza) for 3 days followed by addition of ascorbic acid (AA) or a combination of AA/TGFβ/WY-14643 as indicated for 30 days. (B) Representative micrographs of WY-14643-treated differentiating CDCs which started to aggregate on day 10 and formed organoid/tubular structures on day 20. Scale bar 100 µm (C) Z-stack confocal images of 3D organoids showing expression of MHC, Titin, cTnnT2, MLC, CX43 as indicated, scale bar = 300 µm, unmerged images are shown in Figure S11. (D) The relative mRNA expression of cardiac genes was assessed using qPCR. The mRNA expression was normalized to the geometric mean of Actb and β2M as reference genes and baseline as calibrator; Green stars indicate comparison between baseline and differentiated cells and black stars indicate comparison between differentiated cell groups; * p < 0.05; ** p < 0.01; *** p < 0.001; **** p < 0.0001. (For interpretation of the references to colour in this figure legend, the reader is referred to the web version of this article.)

**Fig. 6:**
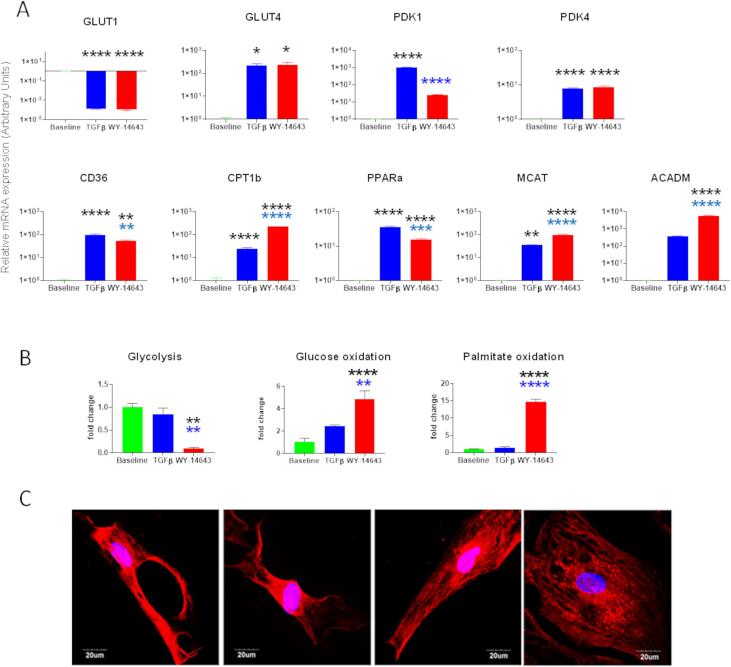
**Metabolic changes after differentiation with TGFβ and WY-14643.** (A) The relative mRNA expression of genes involved in glucose (GLUT1, GLUT4, PDK1 and PDK4) and fatty acid (CD36, CPT1B, PPARα, MCAT and ACADM) metabolism was assessed using qPCR in control, and TGFβ differentiated CDCs with and without treatment with WY-14643. The mRNA expression was normalized to the geometric mean of Actb and β2M as reference genes and baseline as calibrator. (B) Changes in rates of glycolysis and glucose and palmitate oxidation, expressed as a fold change over baseline. Data are presented as mean ± SEM (n = 3) and assessed using an ANOVA with Tukey post hoc test. Black stars indicate comparison between baseline and differentiated cells and blue stars indicate comparison with TGFβ differentiated cells; * p < 0.05; ** p < 0.01; *** p < 0.001; **** p < 0.0001. (C) CDCs differentiated using both TGFβ and WY-14643, stained with Mitotracker® Red CMXRos and showing formation of mitochondrial networks. (For interpretation of the references to colour in this figure legend, the reader is referred to the web version of this article.)

Having concluded that expansion on ColIV and in hanging drops generated P2-CDCs in increased numbers, but that expansion on FN primed cells better for differentiation, we used FN as a substrate for differentiation of P2-CDCs generated via culture on ColIV/HD. To increase differentiation, we adopted a protocol (Fig. 5A) adapted from that of Smits et al (Smits et al., 2009) which we had found to be successful in mouse CDCs (Perbellini et al., 2015) and in addition aimed to stimulate fatty acid oxidation by triggering the PPARα pathway with the agonist WY-14643. Differentiation with the modified TGFβ protocol, both with and without addition of WY-14643, caused the cells to aggregate around day 10 and form organoids after day 20 (Fig. 5B). Immunostaining of organoids, imaged as a Z-stack, showed expression of MHC, cTnnT, MLC, connexin 43 and titin throughout the 3D structure (Fig. 5C). mRNA expression of MHC6, MHC7 and phospholamban (PLN) increased after differentiation with TGFβ. Differentiation with TGFβ plus WY-14643 caused a greater upregulation of MHC7 and cTnnT with increased expression of Serca2a but lower expression of PLN (Fig. 5D). On occasion, organoids generated by treatment with TGFβ and WY-14643 exhibited spontaneous twitching. Differentiation induced a significant decrease in expression of GLUT1 with an associated upregulation of genes associated with glucose and fatty acid oxidation (Fig. 6A). Treatment with WY-14643 induced a small decrease in expression of PPARα and the fatty acid transporter CD36 and a 40-fold decrease in expression of PDK1. Expression of MCAT, CPT1B and ACADM increased 2.5, 10 and 15-fold, respectively (Fig. 6A). Interestingly there was no significant change in metabolism after differentiation but a significant switch from glycolysis to oxidative metabolism with a 10-fold decrease in the rate of glycolysis and a 5-, and 14-fold increase in the rate of glucose and palmitate oxidation, respectively, after treatment with WY-14643 (Fig. 6B). Mitochondrial staining of cells differentiated using TGFβ and WY-14643 (Fig. 6C) showed formation of mitochondrial networks not seen in undifferentiated CDCs (Fig. S10).

## 4 Discussion

*In vitro* isolation and expansion of CDCs on ColIV, with the generation of cardiospheres as hanging drops, resulted in an increased yield of CDCs compared with culture on FN/PDL used in the published protocol (Smith et al., 2007) (Fig. 1A). Cardiospheres formed more quickly in hanging drops than on PDL (Fig. S1b, S1g) and the final spheres were larger (Fig. S1c, S1h), such that cells spent longer within the hypoxic core of the sphere, thought to promote progenitor cell proliferation (Tan et al., 2016). We saw an increased proliferation rate of CDCs on ColIV compared to that on FN (Fig. 1B). ColIV-mediated signalling has been shown to induce proliferation of Leydig cells through intracellular signalling molecules in active forms of focal adhesion kinase and the mitogen activated protein kinase (MAPK) 1/2 (Anbalagan and Rao, 2004) and of pancreatic cancer cells through interaction between the ColIV molecule and integrin receptors on the surface of the cancer cells (Öhlund et al., 2013). ColIV is found within the cardiac stem cell niche (Schenke-Layland et al., 2011), where cells are held quiescent and do not proliferate until required for repair processes, suggesting that other factors within the niche inhibit proliferation. ColIV, but not FN, maintained ESCs in an undifferentiated phenotype, with comparable proliferation rates when cultured on ColIV as on other ECM proteins (Hayashi et al., 2007). We have shown that culture in hypoxia increased CDC proliferation and resulted in cells with higher expression of Oct4, Sox2 and Nanog but that were slower to differentiate than those cultured under normoxia (Tan et al., 2016) as was seen here with cells cultured on ColIV. FN has been shown to promote mesoderm formation in ESCs (Cheng et al., 2013) and induce differentiation through integrin-mediated activation of the MAPK cascade and by a significant increase in the FN receptor, integrin-β5 (Bentzinger et al., 2013, Sa et al., 2014).

We investigated *in vitro* substrate and energy metabolism of isolated cardiac stem/progenitor cells before and after cardiomyogenic differentiation. As expected, CDCs had a highly glycolytic metabolism and the rate of glycolysis was higher in CDCs cultured on ColIV than on FN (Fig. 4A). In comparison to most adult cells, proliferating tumour cells generate energy by glycolysis regardless of the availability of oxygen, known as ‘aerobic glycolysis’ and the Warburg effect (Warburg, 1956), and exhibit greatly enhanced glycolytic flux compared with the non-cancerous cells in the tissue of origin (Moreno-Sánchez et al., 2007). The high level of glycolytic flux provides sufficient ATP for the proliferating cells and glucose degradation generates the necessary biosynthetic intermediates such as ribose sugars for nucleotides and glycerol and citrate for lipids (vander Heiden et al., 2009). In cancer cells, glutamine utilisation has been shown to supplement that of glucose through glycolysis by providing a carbon source for TCA cycle intermediates and, perhaps more importantly, functioning as a nitrogen donor for nucleotide synthesis (DeBerardinis et al., 2007). Glutamine increased doubling time of c-kit + cardiac progenitor cells and promoted survival under conditions of oxidative stress (Salabei et al., 2015). In contrast, we saw very low rates of glutamine utilisation from the media in CDCs before or after differentiation (data not shown). Tardito et al have shown that the glutamine required for glioblastoma growth is not supplied by the circulation but comes from cataplerosis of glucose (Tardito et al., 2015). Interestingly, undifferentiated CDCs cultured on either FN or ColIV had high rates of acetoacetate oxidation (Fig. 4A), which decreased after treatment with 5-Aza. We have found previously that human bone marrow-derived mesenchymal stem cells oxidised acetoacetate 35 times faster than glucose and that this reduced ROS-production 45-fold compared with that seen with glucose oxidation (Board et al., 2017). We hypothesised that this may be a mechanism to reduce the high level of ROS production seen during proliferation and to aid cell survival and maintenance in an undifferentiated state (Suda et al., 2011).

After 5-Aza treatment, CDCs showed increased oxidation of glucose and palmitate (Fig. 4A), associated with a switch in mRNA expression from that of the ubiquitous GLUT1 to the more muscle-specific high-affinity and insulin-sensitive GLUT4, and increased expression of genes involved in fatty acid metabolism (Fig. 3). 5-Aza-treated CDCs on FN demonstrated higher rates of glucose oxidation in comparison to those on ColIV whereas rates of palmitate oxidation were comparable despite a significantly greater upregulation in mRNA expression of genes associated with fatty acid oxidation in cells on FN compared with those on ColIV. ATP production was higher from oxidation of palmitate than from glucose (Fig. 4b), so it may be that whilst treated CDCs on FN had increased their capability to use fatty acid oxidation, compared with those on ColIV, as they had not differentiated to a contractile phenotype they did not need to generate higher levels of ATP. To further probe the upregulation of fatty acid oxidation, we optimized our differentiation protocol by addition of TGFβ (Smits et al., 2009) and stimulated the PPARα pathway by treatment with the agonist WY-14643 which has been shown to upregulate mitochondrial oxidation in induced pluripotent stem cell derived cardiomyocytes (Gentillon et al., 2019). Addition of TGFβ induced the CDCs to aggregate into organoids after 20 days (Fig. 5), as seen by Goumans et al (Goumans et al., 2008), and increased expression of cardiac genes, GLUT4 and genes associated with fatty acid uptake and metabolism, but did not cause a significant increase in oxidative metabolism (Fig. 6). Addition of WY-14643 significantly increased expression of MHC7, cTnnT and Serca2a as well as of genes associated with fatty acid oxidation, compared with that seen in TGFβ-differentiated CDCs, and this resulted in a 5-fold increase in glucose oxidation and a 15-fold increase in palmitate oxidation, compared with undifferentiated CDCs. This was associated with increased expression of cardiac genes, suggesting that this further push towards fatty acid oxidation had increased the cardiomyogenic differentiation of the cells. Although some organoids were seen to twitch spontaneously, the differentiated cells did not mature sufficiently to form functionally competent cardiomyocytes.

## 5 Conclusions

Here we found that the extracellular matrix on which CDCs were expanded determined the balance between proliferation and differentiation, with ColIV promoting proliferation whilst fibronectin primed cells for differentiation to a cardiac phenotype. Undifferentiated CDCs, when highly proliferative, generated high levels of ATP from glycolysis and from oxidation of acetoacetate. As cells differentiated, we observed a decrease in glycolysis, upregulation of oxidative metabolism of glucose and fatty acids and decreased oxidation of acetoacetate. Stimulation of PPARα during differentiation resulted in the cells adopting a metabolic phenotype more akin to that of the adult heart.

## CRediT authorship contribution statement

**Khadijeh Kathy Pakzad:** Conceptualization, Methodology, Investigation, Formal analysis, Writing - original draft. **Jun Jie Tan:** Conceptualization, Methodology, Investigation. **Stephanie Anderson:** Formal analysis. **Mary Board:** Conceptualization, Methodology. **Kieran Clarke:** Conceptualization, Resources, Supervision. **Carolyn A. Carr:** Conceptualization, Methodology, Validation, Resources, Funding acquisition, Project administration, Supervision, Writing - review & editing.

## Declaration of Competing Interest

The authors declare that they have no known competing financial interests or personal relationships that could have appeared to influence the work reported in this paper.
