# Supplementary material for: Metabolic maturation of differentiating cardiosphere-derived cells
Source: Stem Cell Res. 2021 Jul;54:102422. doi: 10.1016/j.scr.2021.102422 (PMC8271094; doi:10.1016/j.scr.2021.102422)
Supplement: Supplementary data 1 [file mmc1.docx]

**Supplementary figures**


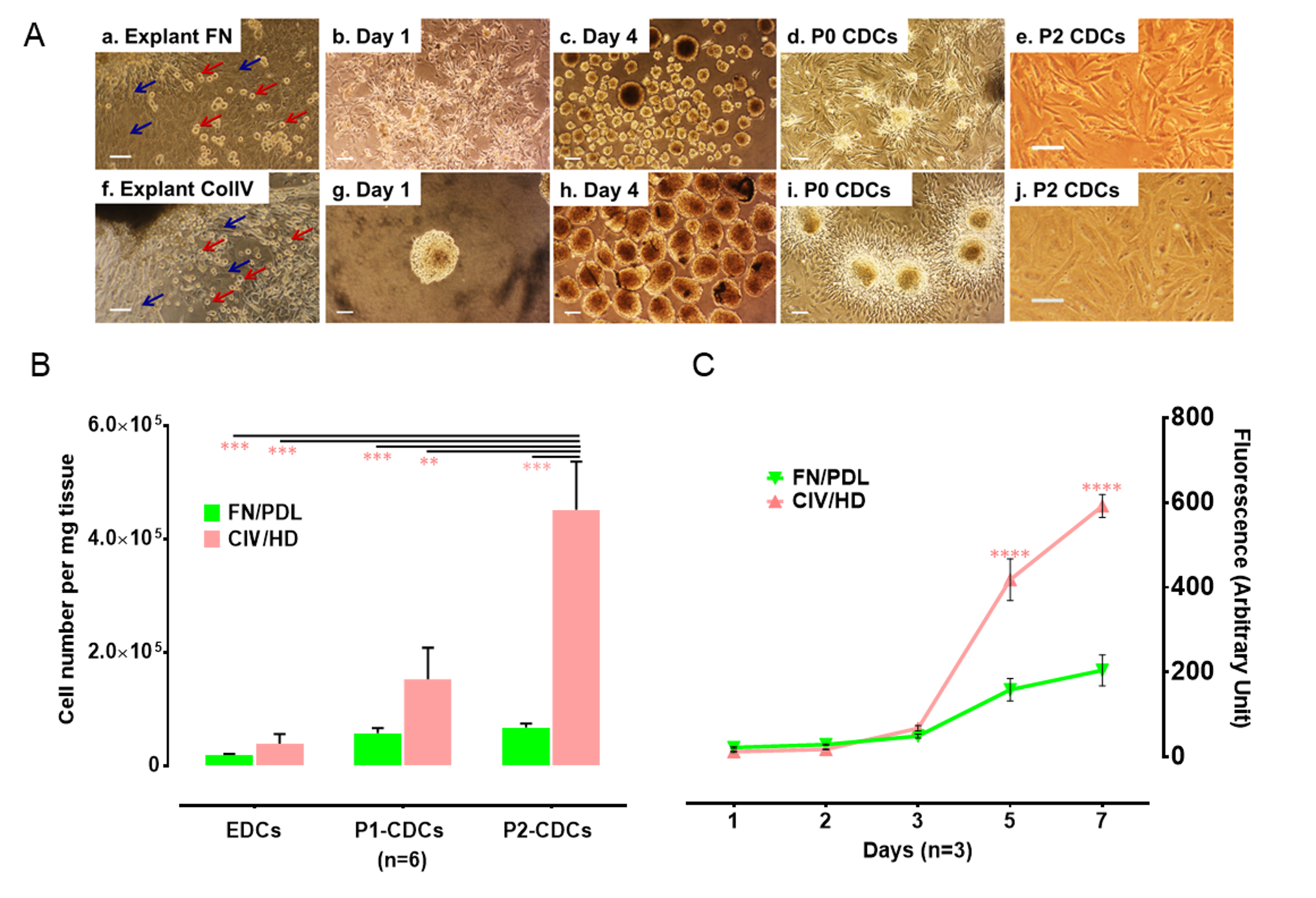


**Figure S1.** **Isolation and expansion of cardiosphere-derived cells (CDCs).** Representative images of isolation and expansion of CDCs on fibronectin/Poly-D-lysine (FN/PDL) (a-e) or collagen IV/hanging drops (ColIV/HD) (f-j). Heart tissue was minced into pieces and explanted on FN (a.) or ColIV (f.) coated Petri dishes. Phase bright cells (red arrows) on top of the stromal-like cells (blue arrows) were confluent by 9-14 days. Explant-derived cells were re-suspended and seeded on PDL-coated 24-well plates (b.) or plated on bacterial dishes as HD (g.) and cultured for 4 days (c and h); CDCs were obtained by culturing cardiospheres on plates precoated with FN (d-e) or ColIV (i-j) until passage 2. Scale bar 100 µm.


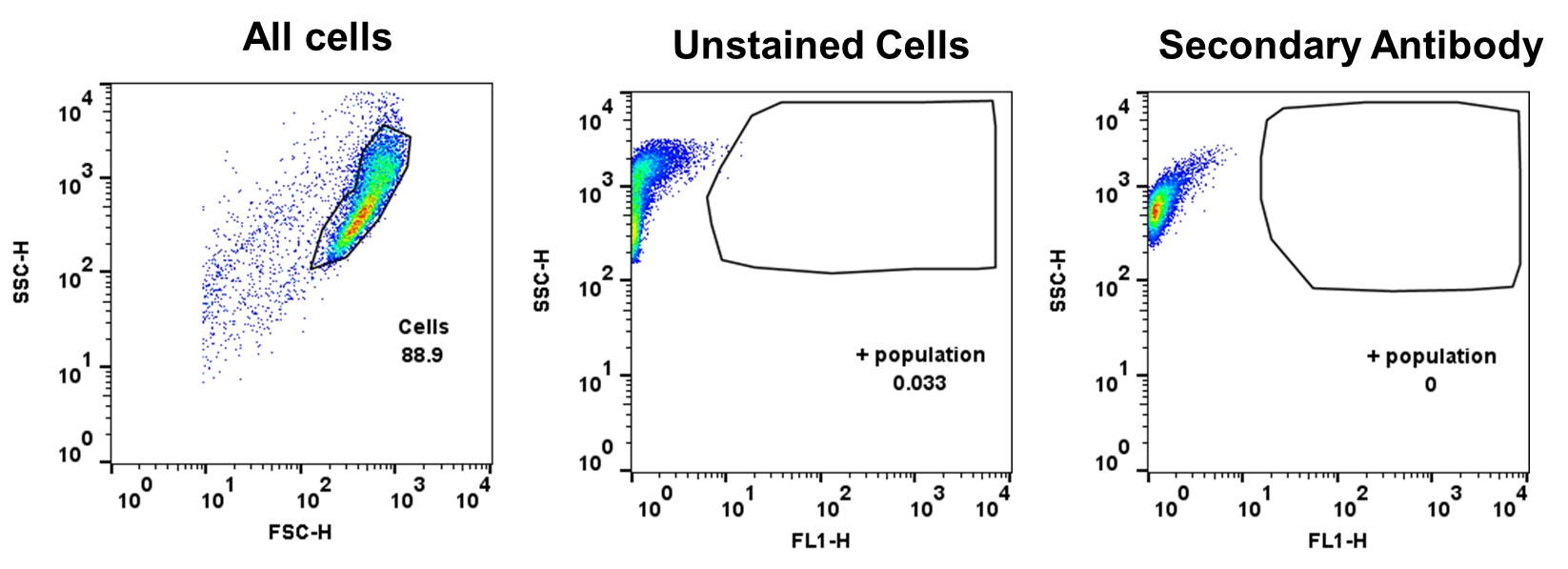


**Figure S2: Flow cytometry analysis.** The total cell population was analysed after gating to exclude debris. Gates were set using populations of unstained cells and cells stained with secondary antibody alone.

**Fig. S3.** Cytotoxicity test of compounds used to treat cells:. P2-CDCs were seeded on FN pre-coated 96-well plates at a cell density of 10,000 cells/cm^2^, left overnight, and treated with media comprising: CEM, CDM-1 containing 5-Aza, CDM-2 containing 10µM or 100µM of WY14643 for 1-, 3- and 7-days and assessed for cytotoxicity test using the LIVE/DEAD®Cytotoxicity assay kit. The level of cytotoxicity was higher in cells treated with 5-Aza than two different concentration of WY-14643 in comparison to the basal medium CEM. Green stars: all groups vs control P2-CDCs, blue stars: 5-Aza day-1 and 3 vs WY14643 day-1 and 3. *: p < 0.05; **: p < 0.001; ***: p < 0.004; ****: p < 0.0001


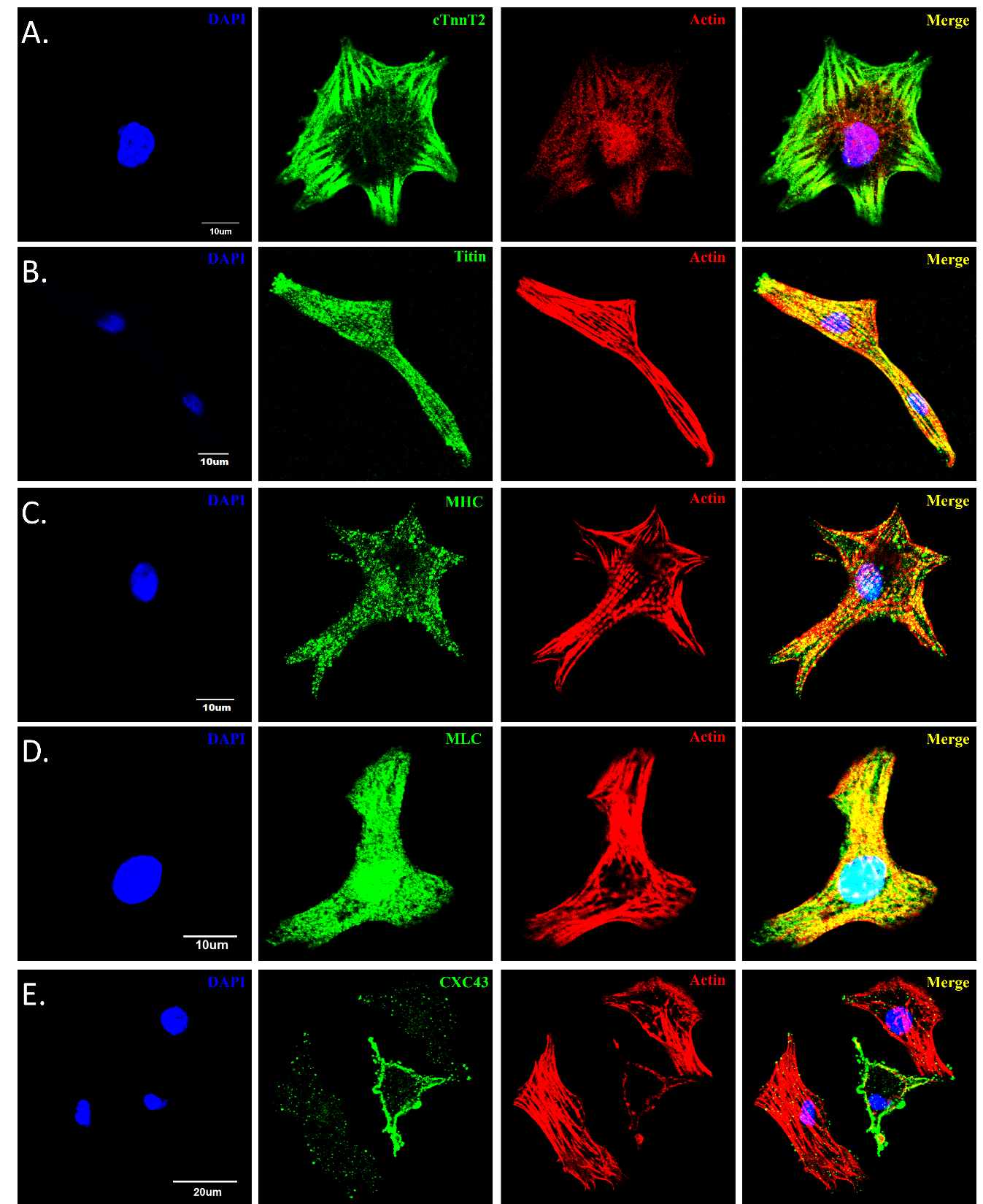


Figure S4: Representative confocal images of positive controls on neonatal rat heart cells. Enzymatically isolated cells from neonatal rat heart and cultured on cover slips for 24 hours were fixed, permeabilized, blocked, stained and visualized by confocal microscopy for cardiac specific cell markers including A. cardiac troponin T2 (cTnnT2) (green), B. Titin (green), C. myosin heavy chain (MHC) (green), D. myosin light chain (MLC) (green), E. Connexin 43 (CXC43) (green) and filamentous actin (F-Actin) (red). Abbreviations: DAPI: 4', 6-diamidino-2-phenylindole. Scale bar 10 μm and 20 μm.

Figure S5: Representative confocal images of negative controls on WY-14643+TGFβ differentiated cells. Differentiated P2-CDCs with WY-14643+TGFβ for 30-days, fixed with PFA, blocked and stained with secondary antibodies only, A. Fluor 488 Donkey anti-Mouse (D@M); B. Alexa Fluor 488 Donkey anti-Rabbit (D@R); C. and D. Alexa Fluor 568 Goat anti-Rabbit (G@Rb). Nuclei were stained with DAPI (blue). Abbreviations: P2-CDCs: Passage 2 Cardiosphere-Derived Cells, transforming growth factor-beta (TGF-β), DAPI: 4', 6-diamidino-2-phenylindole. Scale bar 50 μm.

**Figure S6.** ^14^CO_2_-Capture equipment. (A) The principles of ^14^CO_2_ trapping system and constituent parts shown in (B) comprising filter papers soaked with 40% KOH placed in filter plate and supported with black O-rings (top left); silicon gasket (right), which is pierced above the cell culture wells and placed at the top of cell culture plate (bottom left),

**A.**

**B.**

*
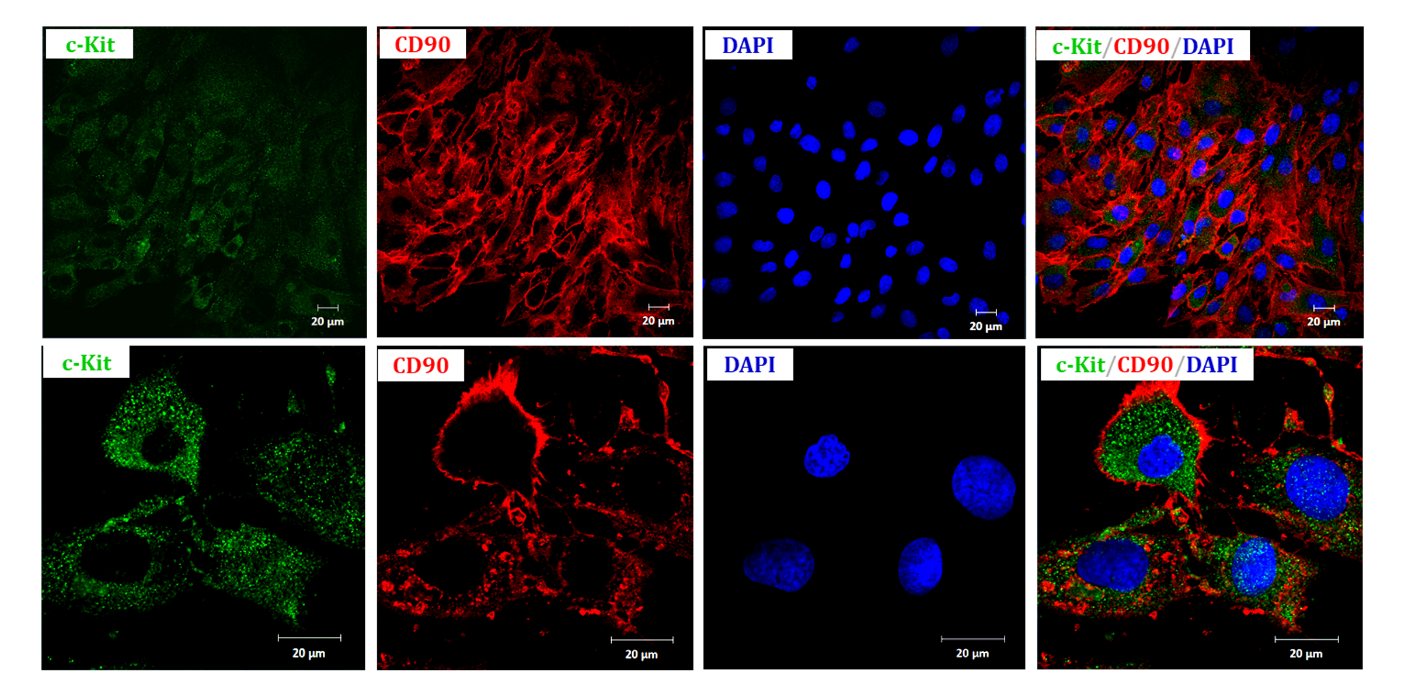
*

**Figure S7: Immunocytochemistry for stem-cell markers**

P2-CDCs isolated grown on FN and as cardiospheres on PDL were seeded on cover slips and fixed with PFA, immuno-labelled with primary then secondary antibodies for c-kit and CD90 and scanned using an Inverted Olympus FV1000 Confocal system.


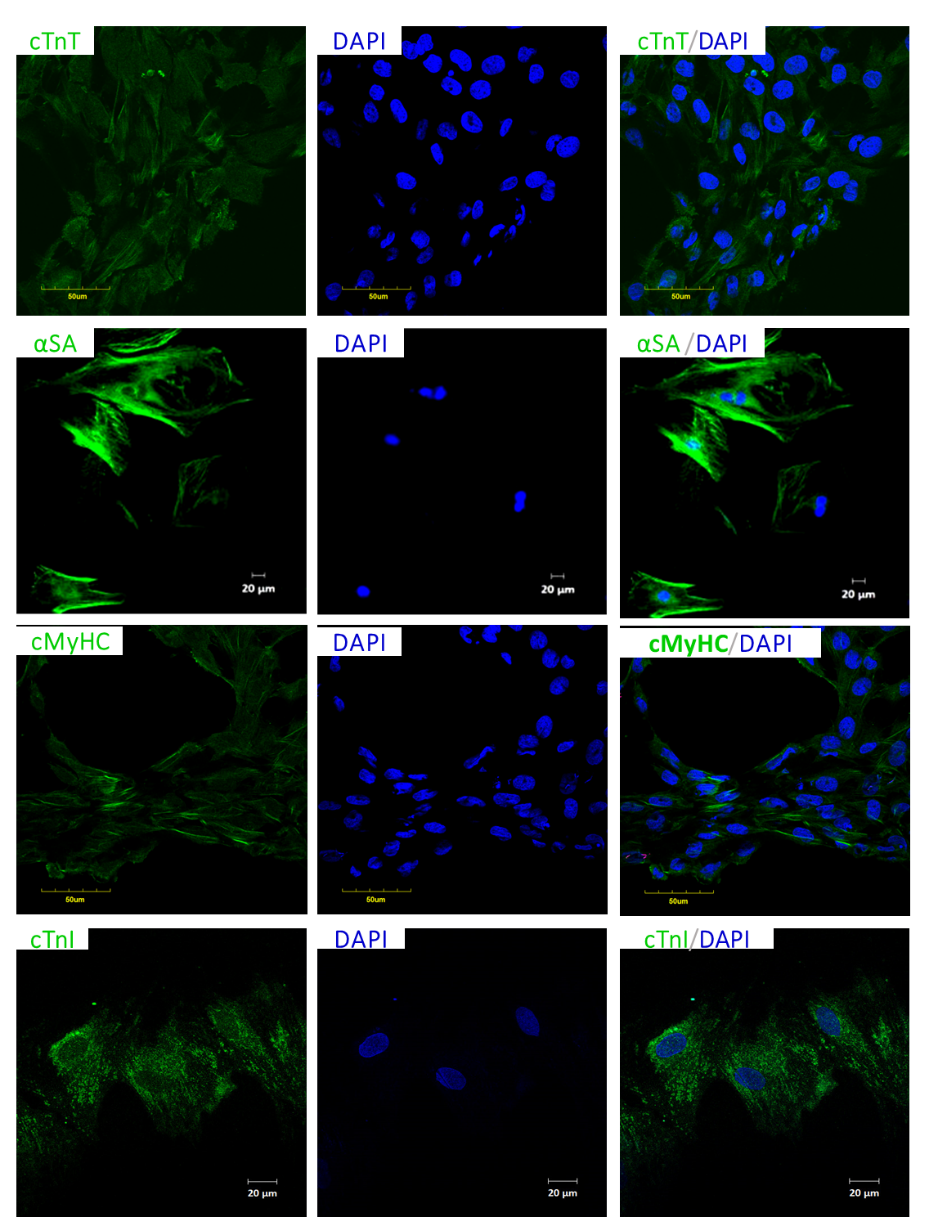


**Figure S8: Immunocytochemistry for cardiac markers**

P2-CDCs isolated grown on FN and as cardiospheres on PDL were treated with 5-Aza. seeded on cover slips and fixed with PFA. Fixed cells were immuno-labelled with primary then secondary antibodies for cardiac troponin T (cTnT), α-sarcomeric actin (αSA), cardiac myosin heavy chain (cMyHC) and cardiac troponin I (cTnI) and scanned using an Inverted Olympus FV1000 Confocal system.

**Figure S9: Western blotting for metabolic proteins**

Western blotting was performed for Peroxisome proliferator activated receptor gamma coactivator 1 alpha (PGC1α), citrate synthase (CISY), α-ketoglutarate dehydrogenase (αKGDH), glucose transporters 1 and 4 (GLUT1, GLUT4 and pyruvate dehydrogenase kinase 4 (PDK4) in control and 5-Aza-treated CDCs. Quantified data are presented as mean ± SEM (n=3), assessed using an ANOVA with Tukey post hoc test. Multicolour stars indicate multiple comparisons between groups and FN and ColIV control; *: p < 0.05;


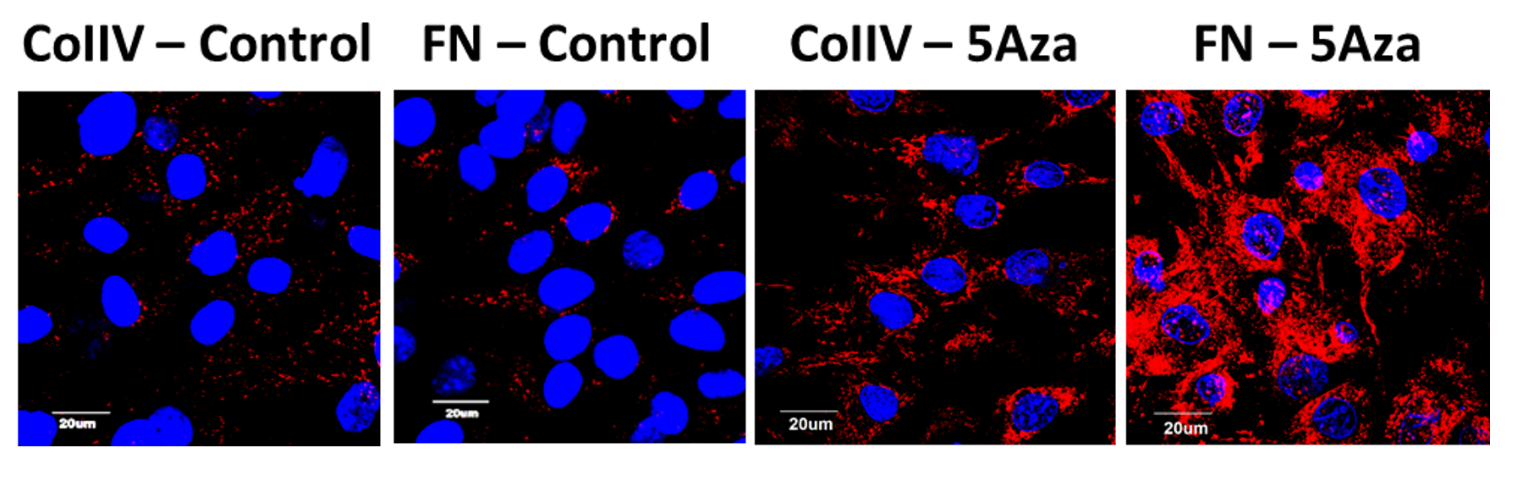


Figure S10. Confocal imaging of control and 5-Aza treated CDCs stained with MitoTracker® Red CMXRos showing increased mitochondrial membrane potential in cells differentiated of FN/PDL.

Figure S11. 3D reconstruction from optical sections of confocal microscopy Z-stack images of organoids formed by differentiation with TGFβ and WY-14643 showing the expression of (A) Titin (green), (B) MHC (green and MLC (red), (C) cTnnT2 (green) and MLC (red) and (D) cTnnT2 (green) and Cx43 (red); with DAPI in blue; scale bars: 300 µm.

**Table S1. *Cell Culture Media***

**Table S2. Primary & secondary antibodies used for flow cytometry & immunocytochemistry**

**Table S3. *Designed primer sequences for cardiac and metabolic related genes***

**Supplementary Methods**

**Western Blotting**

Cells were treated with lysis buffer, removed from the culture surface with a cell scraper and frozen in liquid nitrogen. Cells in lysis buffer were homogenised using a 21 G needle, boiled at 95°C for 5 minutes and centrifuged at 10,000 rpm for 5 min at 4°C, the supernatant was saved. Aliquot of each sample was removed for determination of protein concentration (BCA protein assay kit, Perbio, UK). To the remaining supernatant, 5% β-mercaptoethanol (v/v) was added, followed by boiling for 5 min and storing at 80°C.

Equal amounts of protein (20-40 µg) were diluted in Laemmli loading buffer and loaded onto 12.5% polyacrylamide gels. Gels were run at 120 V for approximately 2 hours. All samples were run in duplicate. Gels were layered with Immobilon-P membrane (Millipore, UK) between extra thick chromatography paper (BioRad, UK), soaked for 30 min in sodium dodecyl sulphate (SDS) transfer buffer and transferred using semi-dry transfer apparatus (BioRad, UK). The membranes were stained with Ponceau S stain (Sigma, UK) to determine successful protein transfer and even protein loading. Blots were washed in TBS-Tween and blocked in 5% (w/v) dried milk in TBS-Tween (25 ml per blot) for at least 1 hour at room temperature. Blots were incubated with the primary antibody at 4°C overnight and then with the appropriate horseradish peroxidase-conjugated secondary antibody. The membrane was covered in enhanced chemiluminescence (ECL) detection solution (Amersham, UK) and sandwiched between sheets of acetate. Membranes were exposed to X-ray film and developed using a Compact X4 automatic X-ray film processor (X-ograph Imaging system, UK). Protein bands were quantified using Un-Scan-It, Version 6.1 (Silk Scientific, USA)
